# Electromechanical analysis of optimal trigger delays for cardiac MRI

**DOI:** 10.1186/1532-429X-16-S1-P73

**Published:** 2014-01-16

**Authors:** Glenn S Slavin, Maggie Fung

**Affiliations:** 1 GE Healthcare, Bethesda, Maryland, USA; 2 GE Healthcare, New York, New York, USA

## Background

Single-phase cardiac MRI acquires data only during a brief period of the cardiac cycle. To avoid motion artifacts, the operator must select a trigger delay that corresponds to a period of minimal cardiac motion, typically at end-systole or mid-diastole. This can be done by inspecting a prior cine scan for quiescent periods. However, because these cardiac phases can vary in temporal position and duration as a function of heart rate, another option should be available if the heart rate at the time of the single-phase scan differs from that during the cine scan. The goal of this work was to analytically determine the optimal trigger delays for cardiac MRI.

## Methods

**Figure 1:**
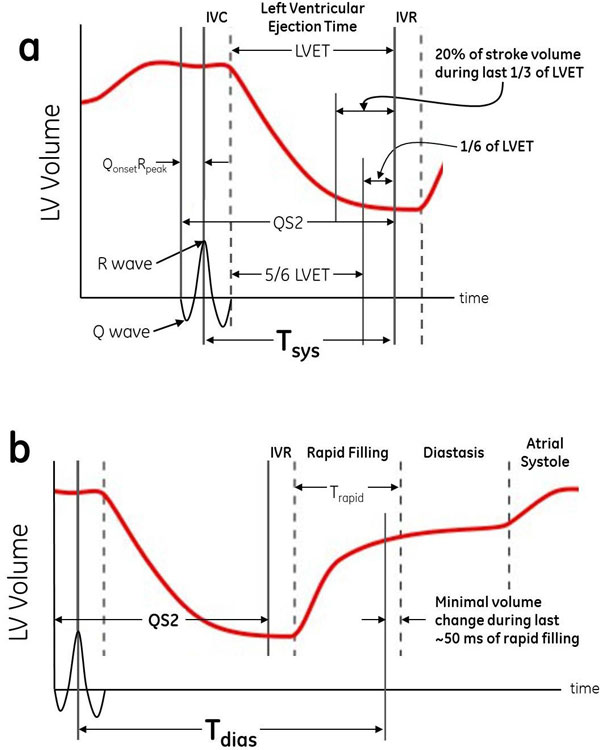
**Left ventricular volume graphs from Wiggers diagram demonstrating optimal trigger delays for single-phase cardiac MRI**. (a) Minimal **systolic** motion begins during the last 1/6 of LVET, when the left ventricular volume change is small. T_sys _= IVC + 5/6 LVET, where LVET = QS2 - Q_onset_R_peak _- IVC. (b) Minimal **diastolic** motion begins slightly before the end of the rapid filling phase. T_dias _= QS2 - Q_onset_R_peak _+ IVR + T_rapid _- 50.

An electromechanical analysis of Wiggers diagram was used to determine the trigger delays (time after R-wave) at which systolic and diastolic quiescence begin (Figure 1).

### Systole

End-systolic quiescence occurs during isovolumic relaxation, but due to the averaging effect of the data acquisition window, reduced heart motion appears slightly earlier. Also, because 20% of the stroke volume is ejected during the last 1/3 of the LV ejection time (LVET) [1], it is assumed here that minimal cardiac motion actually begins during the last 1/6 of LVET. Thus, the optimal systolic trigger delay T_sys _should occur at 5/6 (83%) of LVET. From Figure 1a, T_sys _= IVC + 0.83*LVET, where LVET = QS2 - Q_onset_R_peak _- IVC. Using the substitutions IVC = 40 ms [2], QS2 = 541 - 2.2*HR [3], and Q_onset_R_peak _= 40 ms [4], this gives T_sys _= 423 - 1.826*HR (where HR is heart rate in beats per minute).

### Diastole

End-diastolic quiescence occurs during diastasis, but for MRI scans, sufficiently reduced motion can begin 50 ms earlier [5]. Thus, the optimal diastolic trigger delay T_dias _begins 50 ms prior to the end of rapid filling (T_rapid_). From Figure 1b, T_dias _= QS2 - Q_onset_R_peak _+ IVR + T_rapid _- 50. Using the above substitutions with IVR = 80 ms [6] and T_rapid _= 313 - 0.957*HR [7], this gives T_dias_= 894 - 3.157*HR. These models were compared with ECG-gated short-axis cine scans from 87 adult patients that were retrospectively studied to identify the systolic and diastolic trigger delays at which minimal heart motion began. To assess the efficacy of the equations in a clinical population, only patients with significant akinesis or dyskinesis were excluded. T_dias _was recorded only for patients exhibiting discernible periods of diastasis.

## Results

**Figure 2:**
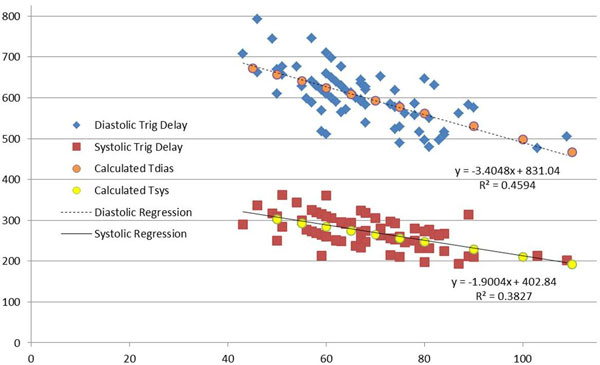
**Plot of trigger delay versus heart rate**. The predicted systolic and diastolic trigger delays show excellent agreement with the regression lines from the clinical measurements.

Figure 2 plots measured trigger delay versus heart rate. Excellent agreement is seen between the regression lines for systolic and diastolic data and the values predicted by the equations.

## Conclusions

Although previous studies have presented general quantitative relationships between various heart phases and heart rate, this work derives recommended trigger delays specifically for use with single-phase cardiac MRI. The models demonstrate good agreement with clinical results and can be valuable for automatically selecting optimal trigger delays when the heart rate varies during an exam or when no reference images are available.

## Funding

N/A
